# Response of vertically-loaded pile in spatially-variable cement-treated soil

**DOI:** 10.1371/journal.pone.0266975

**Published:** 2022-04-13

**Authors:** Tianyue Wu, Zhaohua Sun, Wanxia Tan, Cauderty Munashe Kasu, Jian Gong

**Affiliations:** 1 School of Transportation and Civil Engineering, Nantong University, Nantong, China; 2 Jiangsu Zhongnan Construction Industry Group Company Limited, Haimen, China; 3 College of Transportation Science & Engineering, Nanjing Tech University, Nanjing, China; 4 Guangxi Key Laboratory of Disaster Prevention and Engineering Safety, Guangxi University, Nanning, China; Texas A&M University System, QATAR

## Abstract

Despite the extensive application prospects of piles in cement-treated soil, few studies have explored the ultimate bearing capacity especially in consideration of the spatial variability of cement-treated soil. This study examines the performance of driven piles which were installed inside the cement-treated ground, considering the inherent spatial variability of the cemented soil and the positioning error during piles installation through finite element analyses. The deterministic and random finite element analysis results have shown that the shaft resistance mainly provided the ultimate bearing resistance of piles in cement-treated soil. The spatial variability reduced the global performance of pile installed through a cement-treated soil. The ultimate bearing resistance of the pile inserted in cement-treated soil was controlled by drained condition. Drained ultimate bearing resistance should be used to determine the design working compression load of pile in cement-treated soil.

## Introduction

Pile foundation has been extensively used for soft ground improvement to avoid excessive settlement and differential settlement. The vertical and horizontal bearing capacity of the pile mainly depends on the supporting capacity of the surrounding soil. For piles in cohesive soil, the undrained shear strength of the surrounding soil plays as an important role for the pile foundation design. Ukritchon and Keawsawasvong [1–5] investigated the undrained end bearing capacity of piles in clays involving with anisotropic strengths and the undrained lateral capacity of circular piles, I-shaped concrete piles and rectangular piles.

The strength of the pile cannot be sufficiently utilized under either vertical or lateral loading, and failure caused by soil failure can always occur, which makes it uneconomical for use in soft ground improvement [6]. Pile inserted in cement-treated soil has recently been proposed to improve the foundation bearing capacity [7–9]. Cement-treated soils is usually done by deep-mixing or jet-grouting [10–12]. Both approaches involve installation of overlapping columns, forming walls, layers or blocks. The final structure is expected to have high strength and stiffness, and low permeability compared to untreated soils [13]. However, cement-treated soil exhibits significant random spatial variability [14, 15]. Liu et al. [11] showed that the random spatial variation in properties arising from imperfect mixing and positioning error could significantly affect global strength and stiffness of the cement-treated clay layer. The uncertainty of the soil parameters has significant effect on the bearing capacity of piles [1, 16, 17].

Random finite element analysis has been used to study the effects of spatial variability in soils [18]. Fenton and Vanmarcke [19] presented a fast and accurate method of generating realizations of a homogeneous Gaussian scalar random process in different dimensions. Haldar and Badu [20] investigated the response of a vertically loaded pile in undrained clay considering spatially distributed undrained shear strength. Kasama et al. [21] shown the bearing capacity of cement-treated ground is related to the coefficient of variation and correlation length scale in both shear strength and unit weight. However, despite the extensive application prospects, few studies have explored the ultimate bearing capacity of a pile in cement-treated soil especially considering the spatial variability of cement-treated soil.

An academic exploration was implemented by Finite Element Method (FEM) to simulate the pile inserted in cement-treated soil performances under working loads in this paper. The effect of spatial variability of cement-treated soil on response of ultimate bearing capacity of a pile had been extensively studied. The issue of constitutive behaviour and modelling was first addressed using deterministic analyses, assuming that the cement-treated soil was homogeneous. Random finite element analyses were then conducted to assess the effects of inherent random variation and positioning error. The results in this study can be applied to comprehensively predict the ultimate bearing capacity of piles in cement-treated soil especially considering the spatial variability of cement-treated soil.

### Project profile and results of field load tests

**Fig 1:**
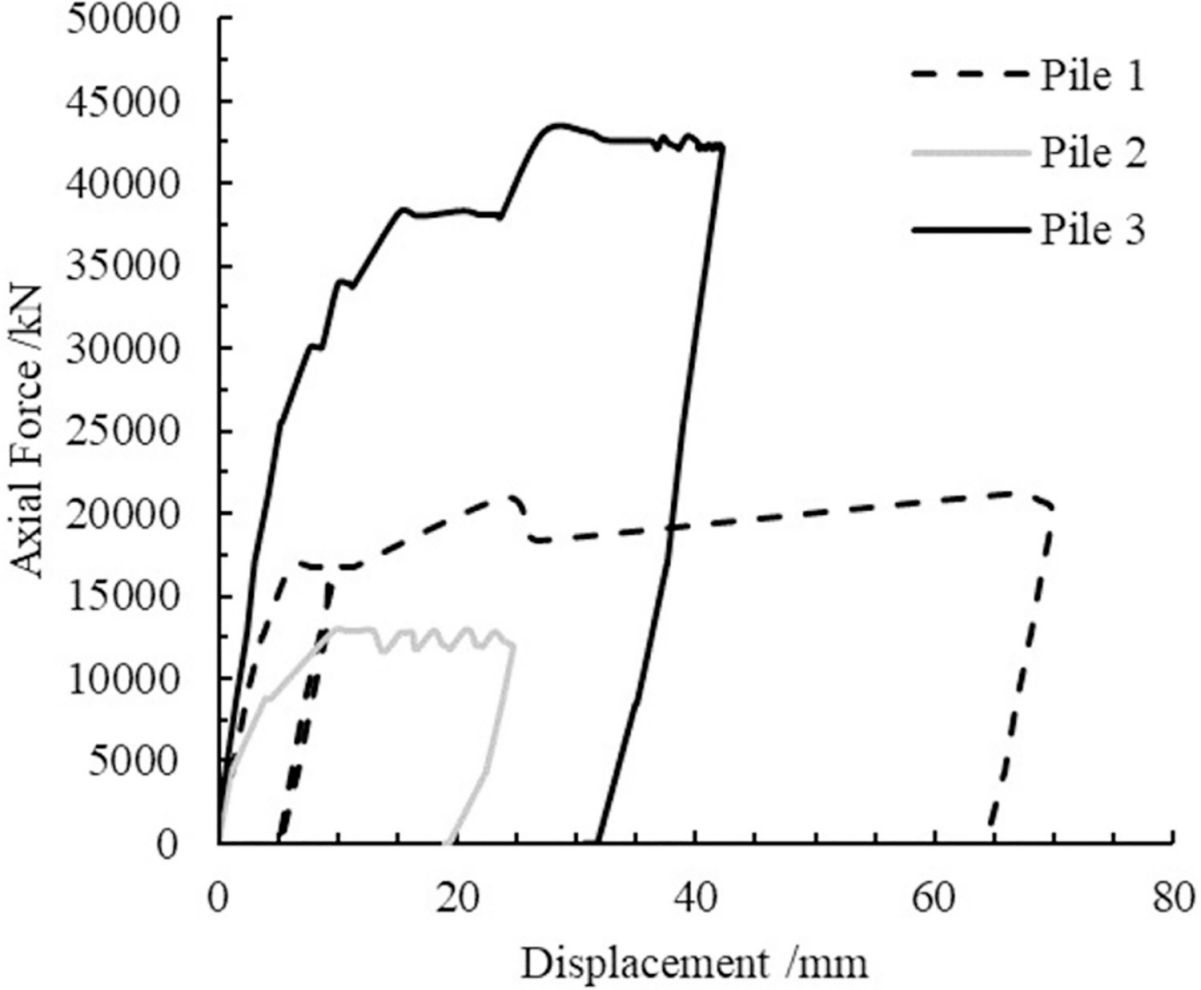
Field pile load tests.

Recently, several driven piles were installed in a cement-treated reclaimed ground in the site of Contract E1003, which is a part of the new Thomson-East Coast Line in Singapore. The whole reclaimed layer (average depth of 40 m) was treated to avoid further consolidation of the soil when the structure is constructed. Several driven piles with diameter of 1.8 m were installed through a cement-treated ground, with its tip embedded in the cement-treated layer. This is rational in the sense that the cement-treated layer has much higher strength than the unconsolidated clay. However, according to the pile loading tests, three out of four piles gave much lower limit loading than the design working compression load. Three field pile load tests were shown in Fig 1. The results of each field pile load test were random. The large variability of the ultimate bearing capacity of the piles is likely to originate from the inherent spatial variability of the cement-treated soil, which is contributed by both uneven distribution of cement binder and the construction error [11, 12, 22, 23]. Provided with only that information, an academic exploration was implemented by Finite Element Method (FEM) to discuss this problem and present solutions. This project was used for reference in following FEM analysis model.

## Deterministic analysis and model verification

### Deterministic analysis

**Fig 2:**
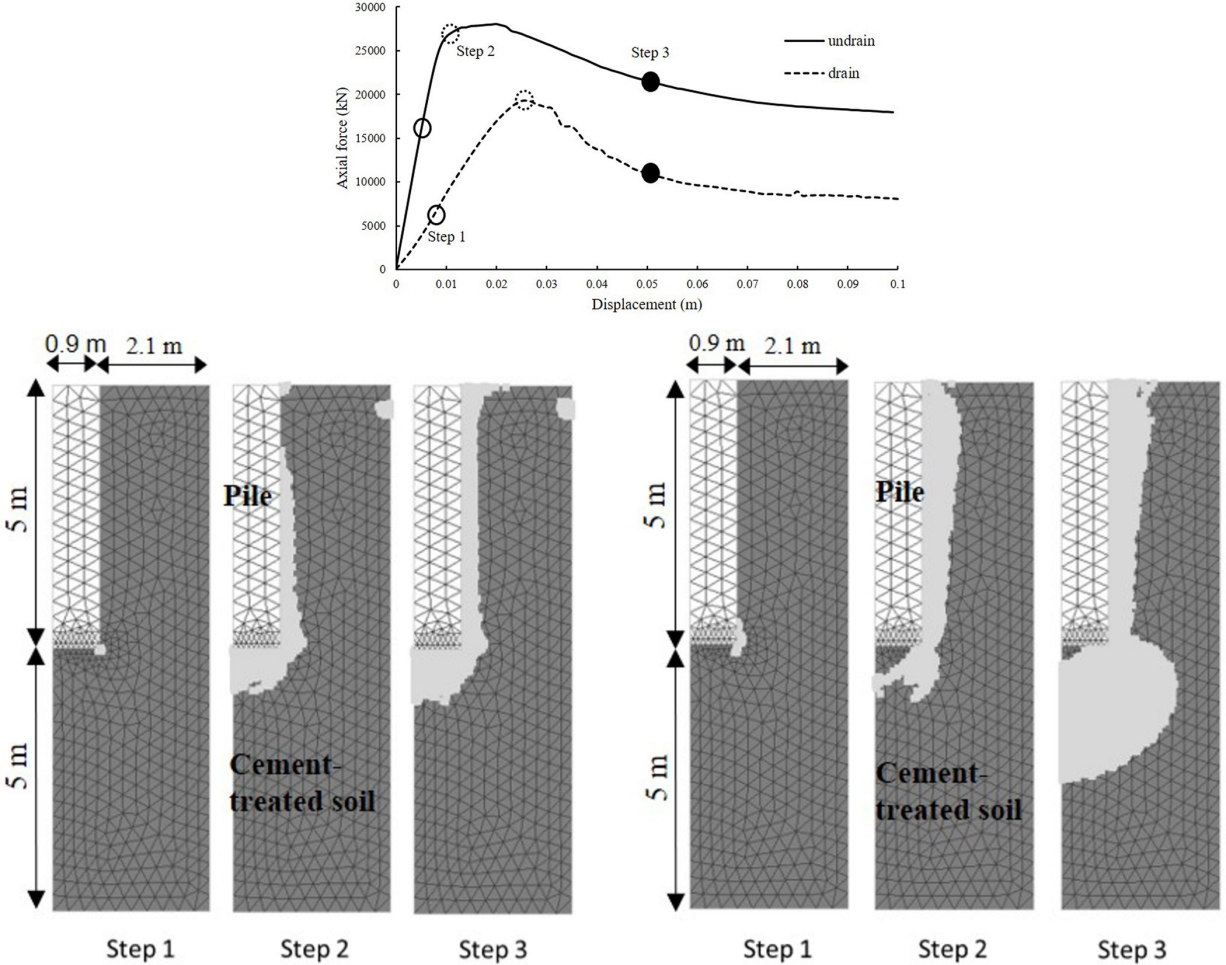
Deterministic analysis: (a) the resistance-displacement curve for pile embedded in cement-treated soil in undrained and drained conditions (b) the corresponding soften status at different step in drained condition (c) the corresponding soften status at different step in undrained condition.

The software was GeoFEA version 9. In deterministic analysis, a two-dimensional axis-symmetric model was first used, with *x* = 0 as the symmetry axis. The rectangular field with width of 0.9 m and height of 5 m was identical to the radius and height of the concrete pile. The cement-treated soil surrounding was modelled by measuring 3 m in radius and 10 m in height. The average mesh size was 0.25 m, as shown in Fig 2. The side boundaries were fixed against normal displacement, while the bottom boundary was fixed in all directions. The top boundary of the pile was moved downwards 0.1 m in displacement control mode to compress the pile model. Preliminary study shows that the distance between the side boundary and the pile boundary was large enough to ensure that the boundary effect was negligible. Totally 938 6-node quadratic triangular elements were used.

The concrete pile was modelled using linear elastic model, given that the strength and stiffness of the concrete pile is much higher than the cement-treated clay surrounding. The cement-treated soil surrounding was simulated using the Cohesive Cam Clay Model (C3 model), which can replicate the strain-hardening and softening behaviour of cement-treated soil [24]. C3 model is an advanced effective stress model developed for cement-treated clay, which can factually model the drained and undrained behaviour of cement-treated soil. C3 model is related ultimately to the unconfined compressive strength of cement-treated soil, which is widely adopted in random finite element analysis studies of cement treated soil [11, 14, 23]. Details of the C3 model, its implementation and validation have been reported by Xiao et al. [24, 25] therefore will not be repeated herein. The model parameters are summarised in Table 1.

**Table 1:** Mode parameters.

Pile (Linear isotropic elastic model)			Cement-treated soil (C3 model)		
Young’s modulus	E_0_(kPa)	3E8	swelling index	κ	0.008
Poisson’s Ratio	υ	0.3	compression index	λ	0.25
bulk modulus of water	K_w_(kPa)	5E6	initial void ratio	e_0_	2.85
bulk unit weight of pile	γ_bulk_(kN/m^3^)	18	friction coefficient	M	2.3
lateral earth pressure	k_0_	0.6	effective Poisson’s Ratio	ν′	0.2
			bulk unit weight of cement-treated soil	γ_bulk_(kN/m^3^)	18
			scale of fluctuation in x direction	SOF_x_(m)	2
			scale of fluctuation in y direction	SOF_y_(m)	2
			unconfined compressive strength	q_u_(kPa)	1000
			coefficient of variation	COV	0
			isotropic degradation parameter	α	2.38
			Initial sensitivity	Si	10

Fig 2(A) shows the drained and undrained resistance-displacement curve for pile embedded in cement-treated soil. As can be observed, the ultimate bearing resistance exhibits significant post-peak softening in undrained and drained conditions. The resistance-displacement curve is almost linear before peak. The drained ultimate bearing resistance was lower than the undrained counterpart. Fig 2(B) and 2(C) indicates the corresponding soften status at different step in drained and undrained conditions. At the pre-peak section (Step 1), only a few points near the corner of the pile yields. At the peak section (Step 2), the ultimate bearing resistance reaches the peak. In undrained condition, all the shaft elements soften and a softening plan formed near the bottom of the pile. In drained condition, most of the shaft elements soften and a much more significant softening plan formed at the bottom of the pile. The post-peak section (Step 3) witnesses the expansion of softening zone.

The difference between the drained and undrained ultimate bearing resistances and their corresponding soften regions at different steps under deterministic analysis indicates that the ultimate bearing resistance of the pile inserted in cement-treated soil were controlled by drained condition. Drained ultimate bearing resistance should be used to determine the design working compression load.

**Fig 3:**
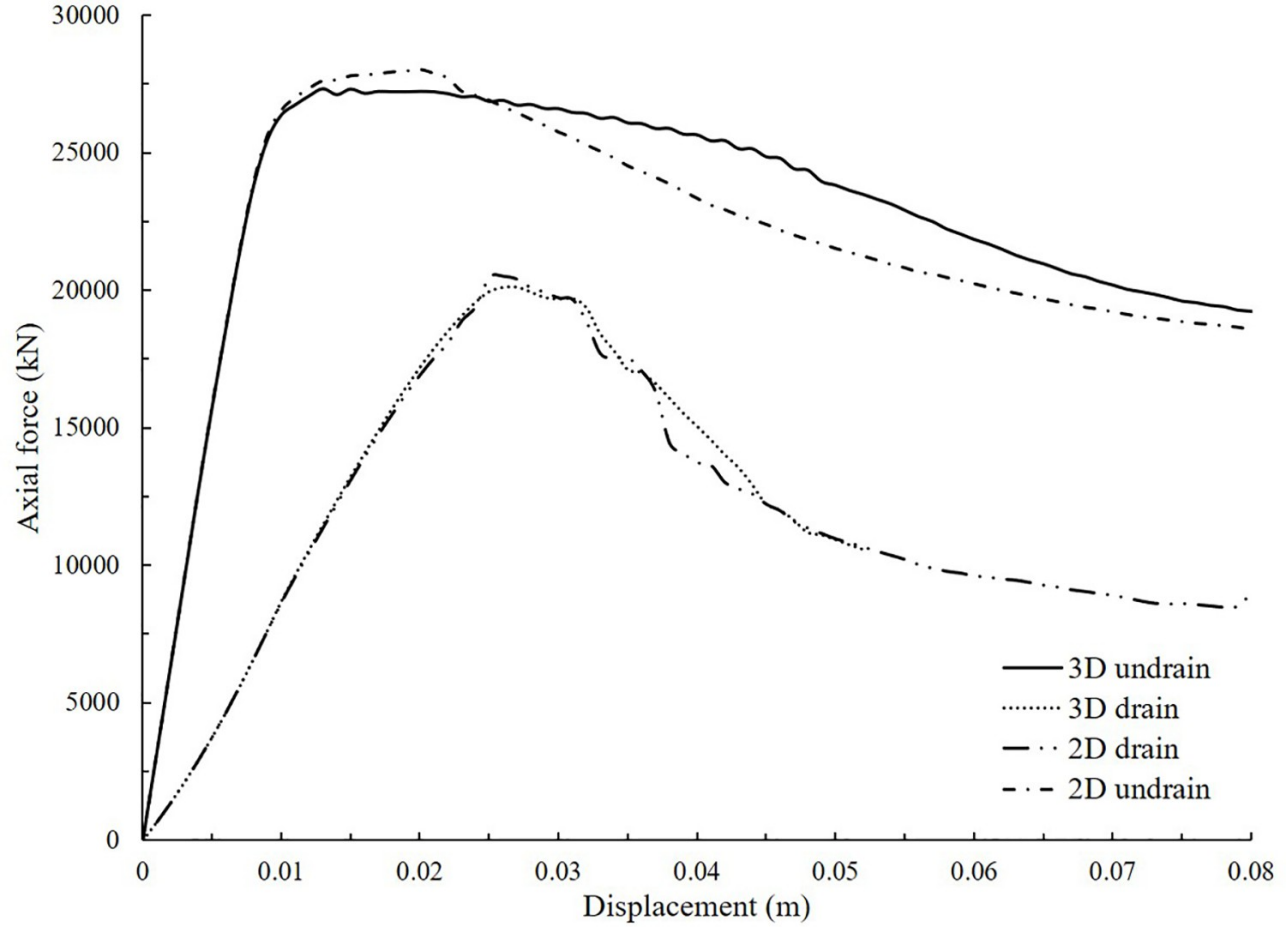
Comparison of 2D and 3D numerical results in drained and undrained condition.

Three-dimensional cylindrical model was then used to represent the homogeneous scenario. The diameter and height of the pile was 1.8 m and 5 m, respectively. The thickness and height of the cement-treated soil surrounded the pile was 2.1 m and 10 m, respectively. The element used was a quadratic tetrahedron element with ten nodes and eleven integration points. The average mesh size was 0.25 m. Totally 8872 nodes and 40459 elements were used. The side boundaries were fixed against horizontal movement while the bottom boundary was fixed in all directions. In the drained or undrained shearing stage, the top boundary of the pile was moved downwards in displacement-control mode. The drained and undrained ultimate bearing resistance curves were consistent with the two-dimensional model results, as shown in Fig 3. As can be observed, the curves under same condition were almost identical, indicating that the dimensionality had little influence on numerical results.

### Model verification

Theoretically, in conventional pile foundation design method, the ultimate bearing capacity of piles in clays was determined by the undrained strength of the undisturbed clay at base level and the average undrained strength of the undisturbed clay over the depth occupied by the pile. The ultimate bearing capacity of a pile in clays can be determined by either analytical or semi-empirical method [26]. Generally, the ultimate bearing resistance (*Q*_*f*_) of a pile in clays is equal to the sum of tip resistance (*Q*_*b*_) and shaft resistance (*Q*_*s*_)

$$Q_{f}=Q_{b}+Q_{s}=A_{b}q_{b}+A_{s}q_{s}$$ (1)

$$q_{b}=c_{u}N_{c}$$ (2)

$$q_{s}=\alpha \bar{c_{u}}$$ (3)

where *A*_*b*_ is the base area; *q*_*b*_ is the ultimate bearing capacity at base level; *A*_*s*_ is the perimeter area of the shaft; *q*_*s*_ is the average value of ultimate shearing resistance per unit area; *c*_*u*_ is the shear strength of the soil mass in the vicinity of the pile base; $\bar{c_{u}}$ is the average undrained strength of the soil over the depth occupied by the pile; *N*_*c*_ is the coefficient of foundation bearing capacity, a value of *N*_*c*_ of 9 is appropriate for piles in clay (i. e. Skempton’s value); *α* is the skin friction coefficient depending on the type of clay, values of *α* can range from around 0.3 to around 1.0.

In the case of piles in cement-treated soil, values of *N*_*c*_ and *α* were calculated according to the FEM deterministic analysis in undrained condition. All of the parameters and constants used were shown in Table 2, which gave a *N*_*c*_ value of 9.0 and a *α* value of 1.0. Then in the FEM analysis the model dimensions and shear strength of the cement-treated soil were changed to further validate the value of *N*_*c*_ and *α*. The result showed that the theoretical result was close to the simulating result of FEM analysis given a *N*_*c*_ value of 9.0 and *α* of 1.0. Therefore these was a good agreement between the theoretical results and the finite element results.

**Table 2:** Bearing capacity coefficient and skin friction coefficient for piles in cement treated soil.

Analysis type	undrain
Simulated ultimate resistance (kN)	27635.86
A_b_(mm^2^)	2.54
A_s_(mm^2^)	28.26
C_u_(kPa)	500
$\bar{C_{u}}$(kPa)	500
q_b_ = N_c_C_u_(kPa)	500N_C_
$q_{s}=\alpha \bar{C_{u}}$	500α
N_C_	9
α	1.0

### Modelling spatial variability of cement-treated soils

The deterministic analysis assumes that the cement-treated soil is homogeneous. However, the cement-treated soil is spatially variable and the presence of spatial variability significantly influence the performance of inserted piles. The coefficient of variation (COV) of unconfined compressive strength of cement-treated soil was assumed 0.4 [11]. The effect of positioning error of pile, input COV and drainage condition on the ultimate bearing resistance of pile was investigated.

The above-mentioned two-dimensional axis-symmetric model was adopted because it was widely used in design of pile and less computational cost is required in random FEA. The spatial variability consists of two parts, i.e. inherent random variation of the cemented soil and positioning error during piles installation. The former is mainly due to the uneven distribution of binder as a result of insufficient mixing [15, 27]. The latter is due to inevitable positioning error during pile installation [11].

The spatial variability is mathematically modelled using a random field. In this study, the modified linear estimation method [28] was used to generate random fields with lognormal marginal distribution. The squared autocorrelation function used in this study is defined as,

$$\rho (\Delta x,\Delta y,\Delta z)=exp[-\pi {\left(\Delta x/\delta_{x}\right)}^{2}-\pi {\left(\Delta y/\delta_{y}\right)}^{2}-\pi {\left(\Delta x/\delta_{x}\right)}^{2}]$$ (4)

where Δ*x*, Δ*y* and Δ*z* are x-, y- and z- distances from the interested point; *δ*_x_, *δ*_y_ and *δ*_z_ are x-, y- and z- scale of fluctuations (SOFs). In this study, *δ*_x_ = *δ*_y_ = *δ*_z_ is adopted to represent a statistically homogeneous material.

On top of the random fluctuation, the positioning error is superimposed. The positioning error stems from the random inclination of deep mixing or jet-grouting shafts [11, 12, 29]. Similarly, the effect of multiple shaft mixing was also modelled using Liu et al.’s [11] approach of prescribing a larger SOF along the alignment of bank of mixing shafts was prescribed. The limit positioning error was set to 0, 375 and 525 mm under drained and undrained condition in random scenarios.

**Fig 4:**
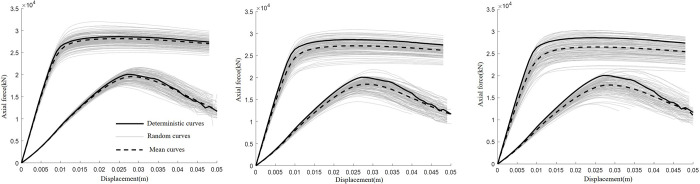
Effect of spatial variability. (a). Maximum deviation of column position 0 mm. (b). Maximum deviation of column position 375 mm. (c). Maximum deviation of column position 525 mm.

Fig 4 shows the 200 random realisations of the reference cases respectively with pile positioning error of 0 mm, 375 mm and 525 mm. As can be observed, most random realisations are below the deterministic curve in each case. According to the law of large numbers, the mean curve in each case is sufficiently close to the true mean value. The deterministic curve is just the result of a perfect situation. However, the mean curve is below the deterministic curve, indicating that it is not safe to use deterministic analysis to determine the ultimate bearing resistance of pile. The difference between the deterministic curve and the mean curve gets bigger as the increase of the pile positioning error. Table 3 shows the mean value (*μ*) and COV (standard deviation/ *μ*) of peak axial force in drained and undrained conditions of these random scenarios. The COV of peak axial force was all less than 0.1. The peak axial force of deterministic analysis in drained and undrained conditions was 2.80ⅹ10^4^ kN and 1.93ⅹ10^4^ kN, respectively.

**Table 3:** Parametric studies in random scenario.

Analysis type	drain			undrain		
Position error (mm)	0	375	525	0	375	525
Average peak value (kN)	1.96×10^4^	1.86×10^4^	1.79×10^4^	2.82×10^4^	2.72×10^4^	2.65×10^4^
Output cov	0.05	0.06	0.09	0.04	0.05	0.07

The results show that the spatial variability reduces the global performance of pile installed through a cement-treated soil. Similar to the deterministic counterpart, the drained result exhibits lower strength than undrained condition. In undrained condition, negative pore pressure was generated in the surrounding cement-treated soil within a short time when the pile under working loads. In drained condition, the dissipation of negative pore pressure for a long time resulted in the decrease of effective stress. The decrease of effective stress of surrounding cement-treated soil and shaft friction in drained condition made the drained ultimate bearing resistance was lower than the undrained one.

Table 3 shows that the positioning error has a significant impact on the ultimate bearing resistance of pile in cement-treated ground. The average peak value and output COV diminishes with increasing amount of positioning errors. The output COV represents the dispersion degree of the axial force results. This is mainly because when the pile tip falls on an untreated zone, the tip resistance would be very low.

### Summary and conclusions

This paper studied the response of vertically-loaded pile in spatially variable cement-treated soil based on FEM. Based on the numerical and theoretical investigations, the following conclusions can be drawn. The foundation design method of piles in cement-treated soils was different from that of piles in clays. Drained ultimate bearing resistance should be used to determine the design working compression load of pile in cement-treated soil. The ultimate bearing resistance of piles in cement-treated soil is mainly provided by the shaft resistance. The positioning error has a significant impact on the ultimate bearing resistance of pile in cement-treated ground. The average peak value and output COV diminishes with increasing amount of positioning errors.

## Supporting information

S1 File This is the S1 File on run deterministic analysis program using GeoFEA. (ZIP)

S2 File This is the S2 File on run spatial variability analysis program with pile positioning error of 0 mm using GeoFEA. (ZIP)

S3 File This is the S3 File on run spatial variability analysis program with pile positioning error of 375 mm using GeoFEA. (ZIP)

S4 File This is the S4 File on run spatial variability analysis program with pile positioning error of 525 mm using GeoFEA. (ZIP)
